# Classification of genomic signals using dynamic time warping

**DOI:** 10.1186/1471-2105-14-S10-S1

**Published:** 2013-08-12

**Authors:** Helena Skutkova, Martin Vitek, Petr Babula, Rene Kizek, Ivo Provaznik

**Affiliations:** 1 Department of Biomedical Engineering, Brno University of Technology, Technicka 12, CZ -616 00 Brno, Czech Republic; 2 International Clinical Research Center - Center of Biomedical Engineering, St. Anne's University Hospital Brno, Pekarska 53, 656 91 Brno, Czech Republic; 3 Department of Chemistry and Biochemistry, Mendel University in Brno, Zemedelska 1, CZ - 613 00 Brno, Czech Republic

## Abstract

**Background:**

Classification methods of DNA most commonly use comparison of the differences in DNA symbolic records, which requires the global multiple sequence alignment. This solution is often inappropriate, causing a number of imprecisions and requires additional user intervention for exact alignment of the similar segments. The similar segments in DNA represented as a signal are characterized by a similar shape of the curve. The DNA alignment in genomic signals may adjust whole sections not only individual symbols. The dynamic time warping (DTW) is suitable for this purpose and can replace the multiple alignment of symbolic sequences in applications, such as phylogenetic analysis.

**Methods:**

The proposed method is composed of three main parts. The first part represent conversion of symbolic representation of DNA sequences in the form of a string of A,C,G,T symbols to signal representation in the form of cumulated phase of complex components defined for each symbol. Next part represents signals size adjustment realized by standard signal preprocessing methods: median filtration, detrendization and resampling. The final part necessary for genomic signals comparison is position and length alignment of genomic signals by dynamic time warping (DTW).

**Results:**

The application of the DTW on set of genomic signals was evaluated in dendrogram construction using cluster analysis. The resulting tree was compared with a classical phylogenetic tree reconstructed using multiple alignment. The classification of genomic signals using the DTW is evolutionary closer to phylogeny of organisms. This method is more resistant to errors in the sequences and less dependent on the number of input sequences.

**Conclusions:**

Classification of genomic signals using dynamic time warping is an adequate variant to phylogenetic analysis using the symbolic DNA sequences alignment; in addition, it is robust, quick and more precise technique.

## Background

The classification of biological sequences (e.g. DNA, RNA, or protein) based on their similarity is a well-known procedure. The similarity between two DNA sequences determined by their evolutionary distance can be used to evaluate the evolutionary relationships of organisms. The evolutionary tree of all living organisms was constructed this way. However, all common methods use a symbolic notation of biological sequences (e.g. symbols A, C, G, T for notation of DNA bases). These methods are usually slow due to high computational complexity. They have low level resistance to errors in the sequences and the application of mathematical evaluation is difficult. The transformation of the biological sequence to a digital genomic signal is a known approach; however there exist only a few methods for subsequent signal analysis [1,2].

One of the possible DNA representations as a genomic signal is a phase determination of complex numbers assigned to the four symbols of DNA record [3,4]. The phase curve of DNA has a characteristic shape for different organisms. This specificity has been proved especially for complete genome [5]. We show that individual genes identified in different species can have a specific character too. For example, the coding segments with different frequencies of symbols have the similar trend. These segments are variously distributed in different sequences because their noncoding sections have different length. The mutations in few nucleotides slightly affect the shape of signals, but the specific trend is preserved. Application of the dynamic time warping (DTW) adjusts positions of these specific sections, but the level of signals remains unchanged. The local differences between sequences can be still compared. This technique is adequate to global multiple alignment of symbolic sequences, but it seems that the dynamic time warping offers wider application than only sequence alignment in comparative genomics [6] and the alignment of DNA in signal representation does not require the substitution matrix. The alignment of symbols depends on individual symbol changes. Therefore, the alignment of coding segments is problematic and must be often corrected manually. Very often, the symbolic sequence alignment is the source of large number of inaccuracies in various applications [7-9].

Phylogenetic analysis represents the typical application of multiple alignments. However, each incorrect assessment causes many imprecisions resulting to incorrect taxonomical classifications [9,10]. This paper presents a new robust method for alignment of biological sequences based on the dynamic time warping applied to genomic signals.

## Methods

### Data description

Actin is a globular structural protein occurring in almost all eukaryotic cells. The highly conserved primary structure between all eukaryotes is one of the most unique properties of actin. The difference in the primary structure of the human and the yeast actin consists in about 5 percent of amino acids. The actin coding genes in different organisms are also very similar. We chose these genes to demonstrate the proposed classification method, because every individual change of a position in a symbolic representation influences a result of mutual similarity. Therefore, the comparison of sequences is complicated.

The actin occurs in six different isoforms differing in a function and a sequence structure. We chose one of them, the alpha actin 1 (ACTA1), which is expressed in a skeletal muscle cells [11]. Ten different organisms were chosen for our comparative study, their characterization is summarized in Table 1. The set of sequences was compiled for demonstration of differences between close organisms and also distant ones. Nine of them are mammalian ACTA1 and a one ACTA1 sequence was obtained from bird tissue. All sequences in this study were obtained from NCBI database (http://www.ncbi.nlm.nih.gov/). The sequences represent complete ACTA1 genes from whole genome shotgun sequences with segments corresponding to mRNA splicing (introns) and they do not reflect only final protein product, but complete genetic information of ACTA1. The length of used sequences is about 2900 ± 200 bp. The corresponding protein products have the same length of 377 aminoacids, without substitutions.

**Table 1:** The specifications of ten DNA sequences from different organisms coding ACTA1

Organism	Chromosome	Accession	Region (sequence position)	Sequence length (bp)
*Homo sapiens *(human, *Hominidae*)	1	NC_000001.10	229566993.. ..229569844	2852
Pongo abelii (Sumatran orangutan, *Hominidae*)	1	NC_012591.1	20230379.. ..20233215	2837
*Macaca mulatta *(Rhesus macaque, *Cercopithecidae*)	1	NC_007858.1	227524284.. ..227527141	2858
*Callithrix jacchus *(Common marmoset, *Cebidae*)	19	NC_013914.1	15737624.. ..15740450	2827
*Mus musculus *(House mouse, *Muridae*)	8	NC_000074.5	126415668.. ..126418637	2970
*Rattus norvegicus *(Brown rat, *Muridae*)	19	NC_005118.2	54081497.. ..54084509	3013
*Sus scrofa (Wild boar, Suidae) *	14	NC_010456.4	65236451.. ..65239197	2747
*Bos taurus *(Cattle, *Bovidae*)	28	NC_007329.5	427530.. ..430286	2757
*Equus caballus *(Horse, *Equidae*)	1	NC_009144.2	68408850.. ..68411788	2939
*Gallus gallus *(Chicken, *Phasianidae*)	3	NC_006090.3	39337938.. ..39340802	2865

### Conversion of DNA sequence to genomic signal

The first step of the DNA classification method is the transformation of DNA symbolic record to numerical form. This procedure is very important, because all biological properties described in the original symbolical form must be maintained in the final genomic signal. We chose the method of conversion representing DNA sequence by the cumulated phase [4].

The transformation technique replaces each of four symbols by its complex value: A [1,j]; C [-1,-j]; G [-1,j]; T [1,-j]. The final cumulated phase corresponds to the value of gradually accumulated sum of angles in complex representation of a DNA sequence and can be calculated for every single position in the DNA sequence by: (1)

(1) $$\Theta_{cum}=\frac{\pi }{4}\left[3\left(n_{G}-n_{C}\right)+\left(n_{A}-n_{T}\right)\right]$$

where n_A_, n_C_, n_G_, and n_T_ are numbers of adenine, cytosine, guanine, and thymine nucleotides in the sequence, from the first to the current location.

**Figure 1:**
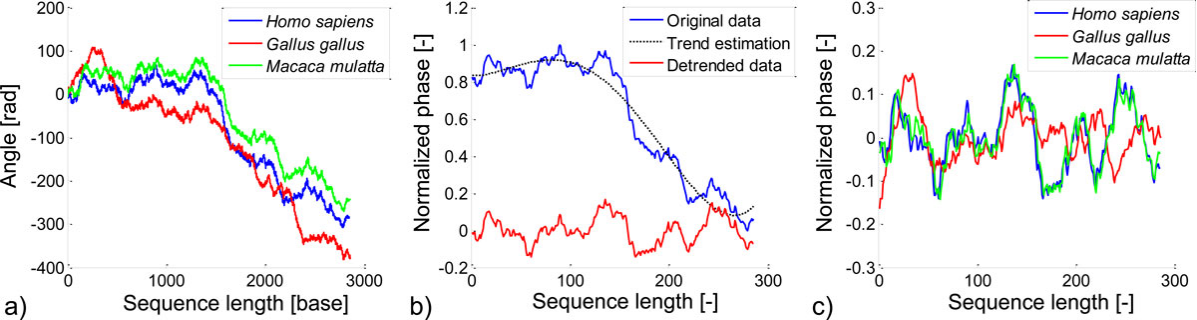
**Genomic signals preprocessing**. a) The record of cumulated phase of the DNA sequences of tree different organisms. b) The principle of detrendization of genomic signal of human ACTA1. c) The resulting preprocessed genomic signals ready for DTW.

The representation of the DNA sequence by cumulated phase maintains the chemical and structural information about original sequence [3,4]. The positional information must be kept to enable the mutual comparison of two sequences. Three curves of cumulated phase for human, rhesus macaque and chicken are shown in Figure 1a. The graphical representation of all 10 genomic signals in a single picture would appear too complex. The pairs of close sequences (human - rhesus macaque) and distant sequences (human - chicken) were selected for demonstration of each step of signals processing. The similarity between human and rhesus macaque cumulated phase is well evident. The curve of chicken ACTA1 cumulated phase is the most different from all other chosen organisms, because other organisms are mammals. However, the certain degree of similarity is still distinguishable.

This fact points to the suitability of the alignment techniques to assess the similarity of sequences. The classical symbolical methods for reconstruction of the similarity diagram from the set of sequences employ the global multiple sequence alignment. These techniques find local similarity in equal positions in sequences and thus are errors inclinable. An approach based on evaluation of similarity between complete signals is expected to be more robust. The resulting distance between genes depends on a trend of each curve.

The dynamic time warping seems suitable for adjustment of derived genomic signals. DTW originally serves for processing of digital signals sampled at defined time instances. In genomic signals, time instances are represented by indexes of nucleotides. The signals based on cumulated phase carry not only useful information, but also noise. Genomic signals preprocessing is necessary to accentuate the position information.

### Genomic signals preprocessing

The preprocessing of genomic signals consists of four steps. The aim is to obtain size adjusted signals suitable for time alignment or, in our case the signals with adjusted size of phase suitable for sequence position alignment. In the first step, the genomic signals were filtered by a simple median filter with a window size of four samples. This signal modification ensures that the values of individual samples do not affect the similarity comparison, but only the significant trend in the signal.

One of the main disadvantages of the symbolic sequence alignment is the requirement to compare all positions. Alignment of the signal representation of genes compares the common segments. Thus, we need to keep only the important components of the signal and we do not need all samples. The second step of preprocessing consists in resampling of signals. The resampling (downsampling) depending on spectral distribution of signal components represents the second stage of preprocessing. The ratio of resampling was estimated based on the power spectrum of genomic signals. The downsampling factor was set to the value of 10. The new sampling frequency (f_s_) of the signal was set with respect to preserve 99.5 % of signal spectral energy.

In the third step, normalization of signals level between 1 and 0 was performed using the linear transform function to ensure the consistent range of values of all signals. Signals of cumulated phase contain significant trend caused by principle of evaluation of the phase. However, the trend does not carry useful information regarding alignment. The trend has nonlinear character and it is necessary to remove it for comparison of local genetic information regardless of the position in the signal. Thus, polynomial detrendisation procedure is the final (fourth) step of genomic signals preprocessing. The estimation of polynomial trend and resulting signal after its removal is shown in Figure 1b. The fourth order polynomial function was found the most suitable for this purpose.

Three signals from Figure 1a were replotted after all mentioned modifications and are shown in Figure 1c. The signals of human and rhesus macaque are almost identical. The signals of human and chicken are more different with a several similar segments, which are shifted in amplitude and position.

### DTW of genomic signals

The dynamic time warping is mostly used for the speech analysis [12]. The same spoken word in the speech of different people has the same meaning (signal has the same shape), but its timing and offset is specific for each person. The dynamic time warping method can adapt the timing and offset of signals [13]. This property can be used for DNA analysis if the DNA sequence is in the form of genomic signal. The time variable is transformed to the nucleotide position and amplitude to the cumulated phase of signals. The word from a spoken language is reflected as a common motive of sequences, most often exons.

We know that ACTA1 protein sequences have 100 % consensus in amino acids. The differences between organisms are only in sequence segments of gene, which are not translated to protein i.e. introns. These segments have dynamic position in sequences; therefore it is necessary to use the methods of dynamic programming, such as DTW. The principle of biological signal alignment using DTW is shown in [14]. This technique aligns sample values based on the minimization of the distance between pairs of samples. Stretching of one or both signals is realized by repeating of the selected samples. The criterion for alignment and repetition of samples is determined by the table of accumulated distances. The values of accumulated distance are calculated from pairwise distances for each pair of samples in accordance with (2).

(2) D(i,j)=min[D(i-1,j-1),D(i-1,j),D(i,j-1)]+d(i,j)

where D symbolizes accumulated distance and d is a value of pairwise distance. The value of accumulated distance D (i, j) is determined by pairwise distance d (i, j) and minimum from the previous values of accumulated distances. This set of accumulated distances for each pair of samples forms a table. The results sequence warping is derived on the basis of minimization of the backward way from the right upper corner to the left lower corner.

**Figure 2:**
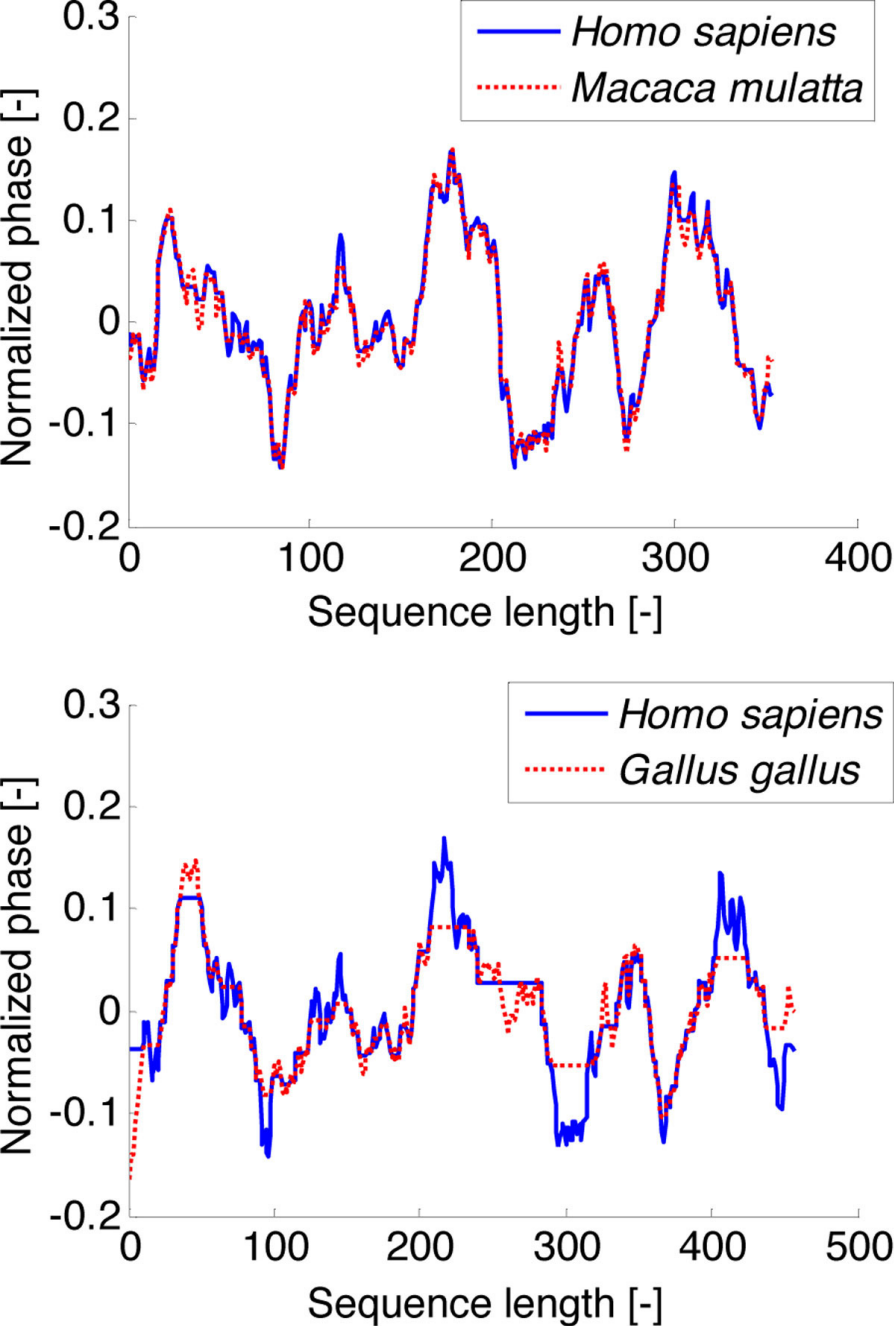
**Genomic signals after DTW**. Upper - the alignment of human and rhesus macaque genomic signals; lower - the alignment of human and chicken genomic signals.

The application of the DTW on genomic signals is shown in Figure 2. It is no surprise that the signals of ACTA1 from rhesus macaque and human are not very different. The use of the DTW in the case of alignment of human and chicken ACTA1 was more necessary. The resulting signals in Figure 2 (lower image) are significantly positionally adapted. The difference between aligned signals is caused by an insufficient amplitude adjustment.

The proposed method is based on alignment of each pair of genomic signals. The described genomic signals preprocessing in combination with a pairwise alignment by the DTW allows mutual adaptation of signals pairs. The resulting genomic signals have generally different lengths than the both original signals.

### Similarity analysis

The similarity of two adapted signals is determined by their Euclidean distance normalized to the length of aligned signals. The distance matrix for cluster analysis is constructed from the mutual similarity values calculated for each pair of signals adapted by the DTW. The distances were normalized to the range <0,1>, because the level of signals pairwise similarity does not have any physical meaning for evolutionary relationships in final dendrogram. These normalized values reflect only the mutual similarity of the set of genomic signals without information about length and amplitude of genomic signals, which is desirable.

## Results

The result of our new approach for sequence similarity analysis is represented by the dendrogram reconstructed from 10 genomic signals of ACTA1. The same dendrogram or phylogenetic tree was reconstructed from the same 10 ACTA1 in classical symbolic form. The method UPGMA (Unweighted Pair Group Method with Arithmetic Mean) was used for phylogenetic tree reconstruction [7]. It allows appropriate comparison with our dendrogram [15]. The evolutionary distance in phylogenetic tree was evaluated by Jukes-Cantor method [16]. The set of DNA sequences was aligned using the global multiple alignment with setting gap penalty equal to 8 for gap open and gap extension too.

**Figure 3:**
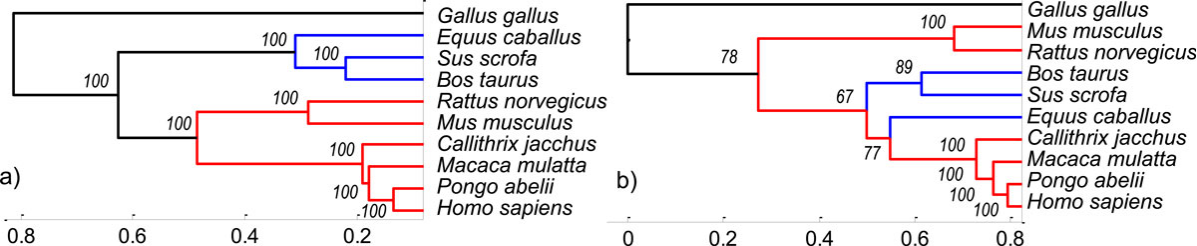
**The results of genomic signals classification**. Similarity analysis of 10 ACTA1 genes presented as a dendrogram constructed from distance values calculated by: a) Euclidean distance between aligned genomic signals after DTW; -b) the evolutionary distance between sequences of symbols aligned by global sequence multiple alignment.

The result trees are shown in Figure 3. The Figure 3a represents the dendrogram reconstructed from genomic signals analysed by the proposed method. The classical phylogenetic tree is shown in Figure 3b.The grouping into clusters in lower tree corresponds to vertebrate phylogeny. Especially the mammals' distribution into two clusters of *euarchontoglires *(supraprimates) and *laurasiatheria *is correct. The mammals' clustering in phylogenetic tree reconstructed by a classical phylogenetic method from symbolic records of sequences is not identical with this.

The robustness of trees is represented by operational taxonomic unit (OTU) Jackknife analysis [17]. The standard technique for testing of tree topology robustness is bootstrapping, but this technique can not be used for genomic signals [18], because our methodology depends on a trend of signals, not only on one position of original sequence. The impact of every single position was eliminated by downsampling and median filtration. The OTU Jackknifing evaluates robustness of the tree as stability of all its nodes. The stability is computed as percentage of occurrence of the each original node in pseudotrees with removing random number of OTUs.

The result values of OTU Jackknifing are evaluated in both trees. The topology of the tree reconstructed by our method (Figure 3a) will not change if we remove any sequences. The standard phylogenetic tree (Figure 3b) does not have sufficiently robust three nodes, because even a single missing sequence can affect multiple alignment and thus the whole phylogenetic analysis. However, the result accuracy depends on setting of preprocessing parameters (i.e. order of polynomial function of trend, window length of median filter and downsampling factor). These parameters are based on analysed sequences, especially on a sequence length and a gene type.

**Figure 4:**
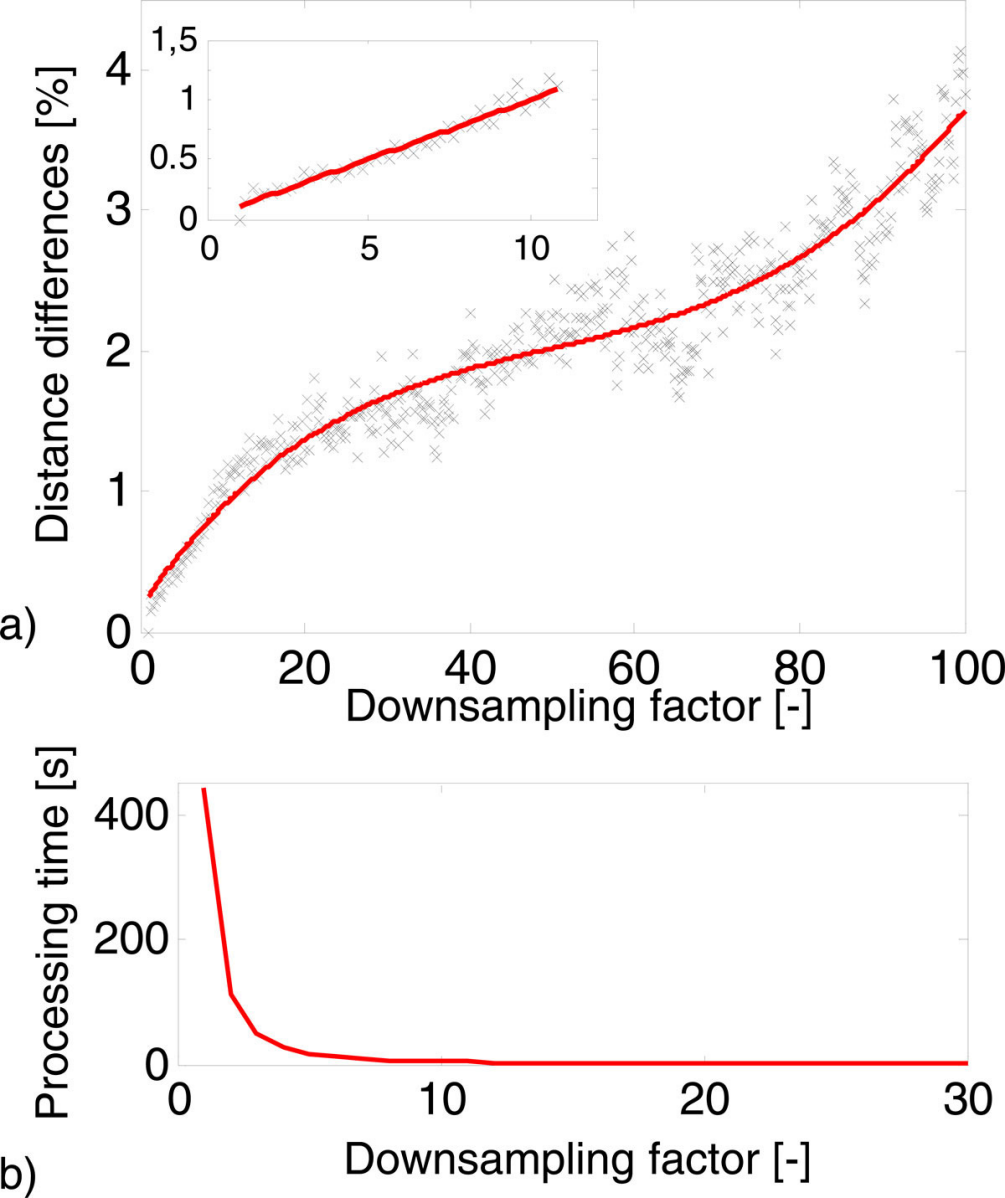
**The influence of downsampling factor of genomic signals**. a) The dependence of change of pair distance on downsampling; b) The dependence of DTW processing time on downsampling.

The utilisation of the proposed method for very different sequences (different genes with different lengths) could cause inappropriate setting of preprocessing parameters and thus errors in similarity evaluation. This problem is similar to setting of parameters of global multiple sequence alignment as scoring matrix or gaps penalties. Low computational load is the greatest advantage of the similarity analysis of DNA represented by genomic signals. Moreover, the signal processing time decreases exponentially with increasing downsampling factor; this fact is presented in Figure 4b. The graph was evaluated on the basis of processing of 10 sequences. The elapsed time for global multiple alignment of this dataset was 49 seconds on standard PC without parallel processing. The time for evaluation of the DTW of 10 genomic signals downsampled by factor 10 decreased to 2.1 second. The signal still contains more than 99.5 % of the useful information after downsampling by ratio 10. In addition, the Figure 4a shows that the increasing downsampling factor has no significant effect on distance differences. The values of distance difference were calculated as the percentage value of sum square differences between distance table calculated for signals with and without downsampling. The trend of dependence between the downsampling factor and the distance differences is almost linear to the value of 10 of downsampling factor (details in Figure 4a), and then changes very slowly increase up to the value of 70. Above the value of 70 of the downsampling factor, the percentage distance differences begin to increase sharply, but all these changes do not exceed 5 percent.

## Conclusions

The results show that the genomic signal processing should have an important place in DNA analysis. Our application of digital signal processing for the similarity analysis is only one of the possible solutions. The main principle of DNA sequences alignment using the genomic signals is an adequate variant to the symbolic DNA sequences alignment; in addition, it is robust, quick and more precise technique. The advantage of alignment of the whole joint sections in signals, not only one position, suggests the use for finding the common motifs or the coding segments. Moreover, the signal character is so specific that the decoding of his features can bring a new perspective on the issues of content and coding of DNA sequences.

## Competing interests

The authors declare that they have no competing interests.

## Authors' contributions

HS, MV and IP conceived the project and wrote the manuscript. HS implemented the algorithm and evaluated the results. MV designed the blocks of signal preprocessing. IP supervised the work. PB and RK ensured the biological aspects of the project. All authors read and approved the final manuscript.

## Declarations

The publication costs for this article were funded by Czech Science Foundation (No. P102/11/1068).

This article has been published as part of BMC Bioinformatics Volume 14 Supplement 10, 2013: Selected articles from the 10th International Workshop on Computational Systems Biology (WCSB) 2013: Bioinformatics. The full contents of the supplement are available online at http://www.biomedcentral.com/bmcbioinformatics/supplements/14/S10.
